# Validating a measure of diminished motivation across the lifespan: Preliminary findings.

**DOI:** 10.1002/alz.089621

**Published:** 2025-01-03

**Authors:** Bonnie M Scott, Amtul‐Noor Rana, Robin C. Hilsabeck

**Affiliations:** ^1^ The University of Texas at Austin Dell Medical School, Austin, TX USA; ^2^ UT Health San Antonio, San Antonio, TX USA

## Abstract

**Background:**

Motivational disturbances are a major harbinger for dementia, being associated with a two‐ to seven‐fold higher conversion rate from mild cognitive impairment. However, there are currently no objective assessment methods for identifying motivational disturbances in older adults (OA). Here, we present preliminary findings from a larger study which aims to validate an objective behavioral measure of effort in OAs by investigating the effects of age, risk, and reward (gain vs. risk trials), and effort type (physical vs. cognitive) on motivated decision‐making.

**Method:**

Two modified versions of the Effort Expenditure for Rewards Task (EEfRT) were developed, including one requiring physical effort (i.e., finger tapping: P‐EEfRT) and one requiring cognitive effort (i.e., working memory: C‐EEfRT). Both tasks included an equal number of gain and loss trials in which participants could choose to expend greater effort to either win or avoid losing points on each trial. Five younger (YA: ages 19‐22) and six older (OA: ages 67‐78) adults completed these tasks along with a cognitive screener and self‐report symptom inventories.

**Result:**

YAs and OAs did not differ in cognitive status nor accuracy scores on the experimental tasks. A significant main effect and interaction of risk/reward magnitude and outcome valence (gain/loss) was found for the frequency of hard task selections on both the P‐EEfRT and C‐EEfRT, suggesting that the decision to exert greater physical and cognitive effort was significantly influenced by the combination of these task parameters in both age groups. A significant age group X outcome valence effect was also observed for both tasks, with YAs exhibiting considerably greater effort expenditure on trials with the possibility of gaining vs. losing points while OAs exhibited a similar pattern of performance for medium to large outcomes on the P‐EEfRT but failed to modulate their responses across task conditions on the C‐EEfRT. Finally, greater self‐reported symptoms of fatigue, apathy, and anhedonia were associated with lower cognitive and physical effort expenditure in the OA group and with slower decision‐making reaction times in the YA group.

**Conclusion:**

Findings suggest that the influence of risk and reward on cognitive and physical effort‐based decision‐making varies across the lifespan.

